# Efficacy of a web-based psychoeducational intervention, Fex-can sex, for young adult childhood cancer survivors with sexual dysfunction: A randomized controlled trial

**DOI:** 10.1016/j.invent.2024.100739

**Published:** 2024-04-07

**Authors:** Kristina Fagerkvist, Kirsi Jahnukainen, Lisa Ljungman, Claudia Lampic, Lena Wettergren

**Affiliations:** a Department of Women's and Children's Health, Karolinska Institutet, Tomtebodavägen 18A, SE-171 77 Stockholm, Sweden; b Centre for Clinical Research Sörmland, Uppsala University, SE-631 88 Eskilstuna, Sweden; c Division of Haematology-Oncology and Stem Cell Transplantation, Children's Hospital, University of Helsinki, Helsinki University Central Hospital, Helsinki, Finland; d NORDFERTIL Research Lab Stockholm, Childhood Cancer Research Unit, Department of Women's and Children's Health, Karolinska Institute and University Hospital Karolinska Institute, Stockholm, Sweden; e Department of Women's and Children's Health, Uppsala University, Akademiska sjukhuset, SE-751 85, Uppsala, Sweden; f Department of Public Health and Caring Sciences, Uppsala University, Box 564, SE-751 22 Uppsala, Sweden; g Department of Psychology, Umeå University, SE-901 87 Umeå, Sweden

**Keywords:** Childhood cancer survivors, Psychoeducation, Randomized controlled trial, Sexual dysfunction, Web-based intervention, Young adults

## Abstract

**Background:**

No web-based interventions addressing sexual problems are available for young adult survivors of childhood cancer.

**Aim:**

This study aimed to test the efficacy of a web-based psychoeducational intervention, Fex-Can Sex, to alleviate sexual problems in young adults treated for cancer during childhood.

**Method:**

This randomized controlled trial tested the effects of a 12-week, self-help, web-based intervention. Young adults (aged 19–40) reporting sexual dysfunction were drawn from a population-based national cohort of childhood cancer survivors and randomized to either an intervention group (IG, *n* = 142) or a wait-list control group (CG, *n* = 136). The primary outcome was ‘Satisfaction with sex life’ assessed by the PROMIS® SexFS v 2.0. Secondary outcomes included other SexFS domains, body image (BIS), emotional distress (HADS), health-related quality of life (EORTC QLQ-C30), and sex-related self-efficacy. Surveys were completed at baseline (T0), directly after the intervention (T1), and three months later (T2). The effects of the intervention were tested using *t*-test and linear mixed models, including intention-to-treat (ITT) and subgroups analysis. Adherence was based on log data extracted from the website system. The intervention included an open-ended question about perceived sexual problems.

**Results:**

No effect of the intervention was found in the primary outcome. Regarding secondary outcomes, the IG reported less vaginal dryness (Lubrication subscale) than the CG at T1 (*p* = 0.048) and T2 (*p* = 0.023). Furthermore, at T1, the IG reported less emotional distress than the CG (*p* = 0.047). Subgroup analyses showed that those with greater sexual problems at T0 improved over time (T1 and T2), regardless of group allocation. Overall, adherence to the intervention was low and participants' activity levels did not change the results. Additionally, some members of the IG reported increased understanding and acceptance of their sexual problems.

**Conclusion:**

The Fex-Can Sex intervention shows potential to improve sexual function, especially among those with greater dysfunction. To increase adherence and effect, we recommend the intervention to be further developed including more tailored content.

**Clinical trial registration:**

ISRCTN Registry, trial number: 33081791 (registered on November 27, 2019).

## Highlights

• Childhood cancer can have a negative impact on sex life.
• This RCT tests the efficacy of a web-based intervention to alleviate sexual problems.
• Few statistically significant differences between the intervention and the control group at post-intervention.
• Adherence to the intervention was overall low.
• Sexuality is recommended to be addressed in childhood cancer follow-up care.

## 1 Introduction

Undergoing cancer treatment during childhood may interfere with important life goals and expectations around sexuality and intimacy when reaching young adulthood. Cancer and its treatment may have a negative impact both physically, due to e.g. hormone disorders or bodily changes, and psychologically (Bober et al., 2013; Zebrack et al., 2004; Sopfe et al., 2020). Commonly reported sexual problems include low interest in having sex (10–36 %), difficulties achieving orgasm (15–32 %) (Bober et al., 2013; Hovén et al., 2021; Zebrack et al., 2010; Bjornard et al., 2020), erectile dysfunction (9–19 %) (Bober et al., 2013; Hovén et al., 2021; Zebrack et al., 2010), vulvar discomfort (19 %), and dissatisfaction with one's sex life (18–20 %) (Hovén et al., 2021; Bjornard et al., 2020). Female survivors report greater impairment on sexual functioning than male survivors (Hovén et al., 2021; Lehmann et al., 2022; Zebrack et al., 2010).

Internet-delivered interventions directed to people with cancer make it possible to reach a large number of participants, regardless of distance from health care services. Most commonly, these interventions focus on symptom management, behaviour change, and emotional health (McCann et al., 2019). It is recommended that web-based interventions should combine information with interactive components, including behaviour change content (Barak et al., 2009; Pingree et al., 2010).

Until now, only a few web-based interventions have addressed sexual dysfunction following cancer, and these have been directed to middle-aged (mean age > 50 years) women with breast or gynaecological cancer (Hummel et al., 2017; Schover et al., 2020) and men with prostate cancer (Wootten et al., 2017; Schover et al., 2012). These interventions included psychoeducational and/or cognitive behavioural components and were evaluated in randomized controlled trials (RCT) (Hummel et al., 2017; Schover et al., 2012; Wootten et al., 2017) or in a pragmatic trial (Schover et al., 2020). The results indicate some positive effects on sexual pleasure with less discomfort during sex (Hummel et al., 2017), reduced erectile dysfunction (Schover et al., 2012, Wootten et al., 2017), increased vaginal lubrication and decreased vaginal discomfort (Hummel et al., 2017, Schover et al., 2020). Additionally, some of the interventions improved self-esteem (Wootten et al., 2017) and body image (Hummel et al., 2017). On the other hand, no effects were observed on orgasmic function (Schover et al., 2020; Wootten et al., 2017; Hummel et al., 2017) or interest in sexual activity (Schover et al., 2020).

To summarize, only a few web-based interventions have addressed sexual dysfunction in cancer survivors, and programmes specifically for young adult survivors of childhood cancer are non-existent. Web-based interventions have the potential to increase access for a large proportion of childhood cancer survivors with sexual dysfunction, and this mode of delivery may be particularly appropriate for those no longer undergoing surveillance. This study aimed to test the efficacy of a web-based psychoeducational intervention targeting sexual dysfunction, the Fex-Can Sex, in young adults treated for cancer during childhood. Furthermore, we aimed to test whether baseline levels of sexual dysfunction and adherence to the intervention affected the outcomes.

## 2 Methods

This study is part of the Fex-Can Childhood, a population-based study with an embedded RCT, described in a study protocol (Ljungman et al., 2020). The current study was designed as a parallel, two-armed RCT with a wait-list control group. The methods and results are presented in accordance with extensions of the Consolidated Standards of Reporting Trials (CONSORT) Statements (Eysenbach, 2011; Montgomery et al., 2018) and the TIDier checklist (Hoffmann et al., 2014).

### 2.1 Participants

**Fig. 1:**
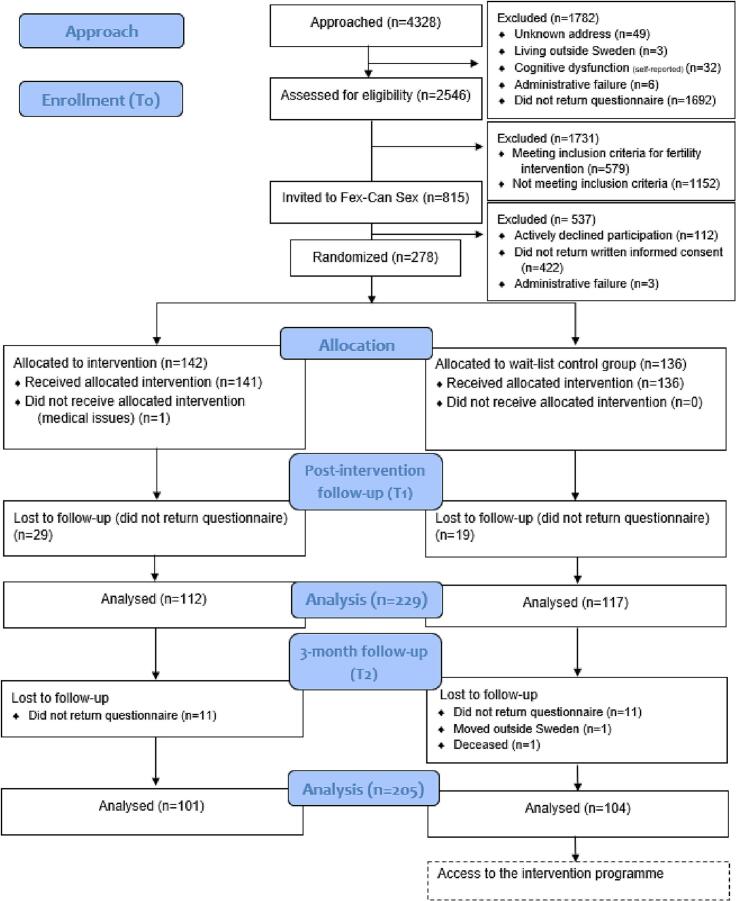
Flow diagram of study participants – CONSORT SPI-2018.

The sample was drawn from a national cohort of young adult (aged 19–40 at the time of the study) survivors of childhood cancer (Hovén et al., 2021) identified through the National Quality Registry for Childhood Cancer (NQRCC) in Sweden. Participants with self-reported sexual dysfunction according to a pre-defined threshold (scoring 0.5 SD below/above the mean in at least one SexFS domain) (Ljungman et al., 2020), were invited to participate in the RCT, see Fig. 1 for flowchart of study participants. Individuals who had moved abroad from Sweden or were deceased were excluded. A total of 815 (444 women and 371 men) survivors of childhood cancer were approached regarding participation in this RCT. Of those, 278 survivors accepted the invitation.

### 2.2 Sample size, randomization, and blinding

Among those reporting sexual dysfunction, approximately 30 % were expected to agree to participate, with an estimated follow-up attrition rate of 15 %. Based on this, we estimated reaching a sample size exceeding 128, which was considered necessary when assuming a power of 80 % and a medium effect size (Cohen's *d*=0.5). Eligible participants who had completed the baseline survey and provided informed written consent were randomized into either the intervention group (IG) or the wait-list control group (CG). Computer-generated randomization was performed by an independent statistician, not directly involved in the trial or data analysis, with an allocation ratio of 1:1, stratified by gender and cancer type (leukaemia/lymphoma, brain tumours, and solid tumours). Due to the study design, it was not possible to blind participants to the group to which they were allocated.

### 2.3 The web-based psychoeducational intervention

The intervention consists of two programmes, Fex-Can Sex and Fex-Can Fertility, which aim to reduce sexual dysfunction and fertility-related distress, respectively. The Fex-Can intervention is guided by self-determination theory (SDT) (Ryan and Deci, 2000), by supporting basic psychological needs: autonomy (i.e., skills for managing emotions and relationships), competence (i.e., knowledge and understanding), and relatedness (i.e., social connection to peers and validation of experiences). Satisfaction with these three psychological needs was expected to result in improved motivation to address potential problems and subsequent behaviour change (Pingree et al., 2010). Both programmes are structured in similar ways, this study reports the effects of the Fex-Can Sex programme. The results from the Fex-Can Fertility programme will be provided in a later report.

Fex-Can Sex is a 12-week, self-help, web-based psychoeducational intervention targeting sexual dysfunction, consisting of six consecutive modules (Table 1), with a new module released every other week. Each module targets a specific aspect of sexuality and includes information, visual illustrations, quizzes, exercises aiming to increase body awareness and acceptance as well as to improve sexual function, and short videos with childhood cancer survivors describing their experiences with the modules' specific topics. Additionally, the intervention includes a discussion forum, described more detailed elsewhere (Gottvall et al., 2022). The intervention is accessible from a computer, smartphone, or tablet. It was designed in accordance with the Medical Research Council's framework for complex interventions (Skivington et al., 2021) and in collaboration with patient research partners (Winterling et al., 2016). To achieve high compliance, participants are informed via email and text message when a new module becomes available.

**Table 1:** Overview of the consecutive modules in the web-based psychoeducational intervention, Fex-Can Sex.

Module		Content	
1	Sexuality – introduction	Sexuality and sexual problems following cancer. Reflections upon sex and one's own sexual habits.	Online moderated discussion forum
2	Lack of desire	How cancer may affect desire, and how to increase desire.	
3	Discomfort and pain Erection	Vaginal pain and discomfort (females) and erection difficulties (males). Exercises and reflections.	
4	Orgasm (females) Orgasm and ejaculation (males)	How cancer may affect the ability to reach orgasm and/or ejaculation. Exercises to enhance pleasure and achieve orgasm.	
5	Relations and sex	Challenges in romantic relationships, dating and meeting a partner. Partner communication.	
6	My body	Possible physical changes following cancer. Exercises to increase acceptance of one's body.	

### 2.4 Wait-list control group

Participants allocated to the wait-list control group received access to the intervention after the post-intervention and follow-up assessments were completed. While participants were on the waiting list, they could request contact with health care, which may or may not include help and support with their sex life.

### 2.5 Measurements and procedure

This study is based on data from surveys and log data from the website. Clinical characteristics (age at diagnosis, cancer type, and treatment modality) were collected from the NQRCC. Cancer treatment intensity was categorized according to the intensity of treatment rating scale (ITR-3) (Kazak et al., 2012; Hovén et al., 2021). The intervention was performed in two batches for the intervention group (December 2019 to April 2020) and once for the wait-list control group (August to November 2020).

Both groups completed a survey at baseline (T0), directly after the intervention (T1), and three months later (T2) (August 2019 to August 2020). Participants were able to complete the survey via paper or the web. Two written reminders were sent to non-responders. Participants received two cinema tickets after answering each survey.

#### 2.5.1 Primary outcome

The primary outcome was the domain Satisfaction with sex life measured by the Patient Reported Outcome Measurement System (PROMIS®) Sexual Function and Satisfaction Measure version 2.0 (SexFS) (Weinfurt et al., 2015). Item response theory was used to calculate domain scores, which were transformed to a T-score metric, where 50 (1SD = 10) represents the mean for the general sexually active US population. The SexFS has shown satisfactory validity and reliability (Sopfe et al., 2021; Weinfurt et al., 2015). The Swedish version has demonstrated adequate psychometric properties (Hovén et al., 2023).

#### 2.5.2 Secondary outcomes

The secondary outcomes regarding sexual function include three generic domains of SexFS: Interest in sexual activity, Orgasm pleasure, and Orgasm ability. In addition, five body part specific (four for women, one for men) domains were selected: Vaginal lubrication, Vaginal discomfort, Vulvar discomfort – clitoral, Vulvar discomfort – labial, and Erectile function.

Secondary outcome measures also include the Body Image Scale (BIS) to assess body image disturbances following cancer (Hopwood et al., 2001); the Hospital Anxiety and Depression Scale (HADS) to assess emotional distress (Zigmond and Snaith, 1983) and the well-established Health-related quality of life measured using the summary score of EORTC QLQ-C30 version 3.0 (Aaronson et al., 1993; Giesinger et al., 2016).

In addition, sex-related self-efficacy was measured by means of six study-specific items. These items measured the participants' ability to handle situations, emotions, and thoughts related to their sexuality. A total mean score was calculated, with higher values indicating higher levels of sex-related self-efficacy.

##### 2.5.2.1 Adherence to the intervention

Adherence to the intervention was measured from log data extracted from the website system. Participants were categorized based on their level of adherence: high, low (IG participants), or control (CG participants). High activity was defined as fulfilling criteria for both general activity (opened ≥3 modules and spent ≥20 min in the intervention) and at least one criterion for interactive activity (spent ≥3 min in the discussion forum, written ≥1 post, or answered ≥50 % of the quizzes) (Micaux et al., 2022). Low activity users included those who had never logged in.

##### 2.5.2.2 Self-perceived change

At T1, members of the IG rated their level of sexual problems compared to before they entered the intervention on a 3-point Likert scale: “Improved”, “No change” and “Worsened”. Furthermore, at the end of the intervention, participants had the opportunity to comment on their sexuality and sexual problems.

### 2.6 Statistical analysis

To test the effects of the web-based intervention compared to the control group, intention-to-treat (ITT) analyses were used for all primary and secondary outcomes. Missing data in SexFS was handled with item response theory (IRT) according to the established PROMIS methodology, i.e. an assumption score based on possibility models (Weinfurt et al., 2015). Missing data in other measures was handled as follows: single missing items were imputed according to the individual's mean on the scale/subscale, given that at least half of the items had been answered. If less than half of the scale/subscale had been answered, no imputation was performed. Sociodemographic and clinical characteristics of the participants in the IG and CG were compared at baseline by gender using Student's *t*-tests and Chi-square tests, as appropriate. Effects of the web-based intervention were tested using between-group comparison (Independent *t*-test). Cohen's *d* for Effect size were used to determine changes of clinical importance, with values of 0.2, 0.5, and > 0.8 considered as small, medium, and large, respectively (Cohen, 1988). Sensitivity analyses was performed, for statistically significant differences with t-tests, using multiple linear regression to explore the potential influence of baseline level and group allocation.

Linear mixed models (LMM) analysis with a subject-specific random intercept was used for subgroup analysis. An advantage of LMM is that the method minimizes the loss of information due to missing values (Hesser, 2015). For the SexFS domains, LMM were used to analyse interaction effects of time and level of sexual dysfunction at baseline (more sexual dysfunction defined as ≥1SD from the mean of the US population). Subgroup analysis was also performed by level of activity in the intervention (low level of activity, high level of activity and CG).

All statistical analyses were performed using SPSS Statistics version 28 (IBM Corp., Armonk, N.Y.) and lme4 and lmerTest package in R version 4.2.1. All tests were two-tailed and a *p*-value of ≤0.05 indicated statistical significance.

### 2.7 Ethical considerations

Ethical approval was obtained from the Regional Ethical Review Board in Stockholm, Sweden (Dnr: 2015/1609-31; 2018/2688-32; 2019/01066). Written informed consent was obtained from all participants in the study. To gain access to the intervention, participants logged in with their email and their own chosen password.

## 3 Results

### 3.1 Study participants

A total of 278 participants, 165 women and 113 men, were included in the RCT. Detailed characteristics of participants at randomization are presented in Table 2; sociodemographic and clinical characteristics did not differ between the groups (data not shown).

**Table 2:** Characteristics of the study population at randomization.

	Intervention group		Control group	
	Women *n* = 83	Men *n* = 59	Women *n* = 82	Men *n* = 54
	No. (%)	No. (%)	No. (%)	No. (%)
Sociodemographics				
Age at study, years				
Mean (SD)	29.4 (6.0)	30.5 (6.2)	28.8 (5.9)	28.9 (6.3)
Country of birth				
Sweden	80 (96.4)	57 (96.6)	78 (95.1)	53 (98.1)
Other	3 (3.6)	2 (3.4)	4 (4.9)	1 (1.9)
Education				
Elementary	2 (2.4)	0 (0)	3 (3.8)	3 (5.5)
Upper secondary	25 (30.1)	27 (46.6)	29 (36.3)	28 (51.9)
University	52 (62.7)	27 (46.6)	43 (53.8)	21 (38.9)
Other	4 (4.8)	4 (6.8)	5 (6.3)	2 (3.7)
Occupation				
Working/studying	66 (79.5)	54 (91.5)	69 (85.2)	45 (83.3)
Other^a^	17 (20.5)	5 (8.5)	12 (14.8)	9 (16.7)
Partnered	58 (69.9)	27 (46.6)	56 (68.3)	19 (35.2)
Have children	30 (36.1)	15 (25.9)	30 (37.0)	9 (16.7)
Sexual orientation				
Heterosexual	67 (82.7)	54 (91.5)	68 (84.0)	50 (92.6)
Non-heterosexual^b^	14 (17.3)	5 (8.5)	12 (14.8)	4 (7.4)

Clinical characteristics				
Age at diagnosis, years				
Mean (SD)	7.1 (5.8)	8.8 (5.3)	7.2 (5.3)	8.2 (5.3)
0–5	45 (54.2)	17 (28.8)	35 (42.7)	22 (40.7)
6–12	15 (18.1)	24 (40.7)	29 (35.4)	15 (27.8)
13–17	23 (27.7)	18 (30.5)	18 (21.9)	17 (31.5)
Time since diagnosis, years				
Mean (SD)	22.2 (7.3)	21.7 (7.7)	21.5 (7.4)	20.7 (7.8)
Range	3–37	4–36	6–36	5–35
Type of cancer^c^				
Haematological	39 (47.0)	28 (47.5)	38 (46.3)	28 (51.9)
CNS tumours	20 (24.1)	18 (30.5)	20 (24.4)	16 (29.6)
Solid tumours	24 (28.9)	13 (22.0)	24 (29.3)	10 (18.5)
Treatment modality				
Chemotherapy	71 (85.5)	50 (84.7)	74 (90.2)	49 (90.7)
Surgery	30 (36.1)	21 (35.6)	33 (40.2)	22 (40.7)
Radiotherapy	25 (30.1)	23 (39.0)	32 (39.0)	22 (40.7)
Stem cell transplantation	7 (8.4)	7 (11.9)	7 (8.5)	7 (13.0)
Intensity of treatment^d^				
Least intensive	5 (6.0)	3 (5.1)	9 (11.0)	3 (5.6)
Moderately intensive	42 (50.6)	29 (49.2)	36 (43.9)	22 (40.7)
Very intensive	23 (27.7)	16 (27.1)	22 (26.8)	19 (35.2)
Most intensive	13 (15.7)	11 (18.6)	15 (18.3)	10 (18.5)
Relapse/second malignant neoplasm				
Yes	8 (9.6)	6 (10.2)	8 (9.8)	8 (14.8)

CNS, central nervous system.

a Includes unemployment, sick-leave, parental leave.

b Includes not knowing, being pansexual, queer, or asexual.

c According to the International Classification of Childhood Cancer (ICCC-3).

d According to the Intensity of Treatment Rating (ITR-3).

The response rate was 82 % at T1 and 74 % at T2. There were no differences between responders and non-responders regarding sexual function at T0. At T1, female responders and non-responders did not differ in any sociodemographic or clinical characteristics (age at diagnosis, time since diagnosis, cancer type, treatment modality, or treatment intensity). Among males, the responders were to a larger extent working/studying (99 % vs 73 %, *p* = 0.016), and partnered (45 % vs 24 %, *p* = 0.044) than the non-responders.

### 3.2 Use of the intervention

Of the 142 participants randomized to the IG, 42 % (n = 59) reached the level of use defined as high activity, 51 % (*n* = 73) had an activity level corresponding to low and 7 % (*n* = 10) had not logged into the intervention at all. Women spent significantly more time in the intervention with an average of 49.2 min (SD 53.7 min, median 31.1 min) than men at 31.0 min (SD 41.8 min, median 14.3 min, *p* = 0.025). An overview of the percentage of participants opening each module and the amount of time spent in the respective module, by gender, are presented in Table 3. In the IG, 28 % (*n* = 40) of participants had spent at least 3 min in the discussion forum and 11 % (*n* = 15) of participants had posted at least once. More women answered ≥50 % of the quizzes than men (74 % vs 49 %, *p* = 0.003).

**Table 3:** Overview of percentage of participants opening each module and the amount of time spent in the respective module by gender.

Modules	Women				Men			
	Opened (%)	Time (minutes)			Opened (%)	Time (minutes)		
		Mean	Median	Range		Mean	Median	Range
#1 Sexuality	99	16.3	10.8	0–81.6	83	12.4	5.9	0–66.6
#2 Lack of desire	77	9.4	5.2	0–59	61	6.2	0.7	0–83.3
#3 Discomfort and pain	57	6.5	2.0	0–37.3	N/A	N/A	N/A	N/A
#3 Erection	N/A	N/A	N/A	N/A	51	5.6	0.0	0–70
#4 Orgasm	58	3.1	0.8	0–29.1	N/A	N/A	N/A	N/A
#4 Orgasm/ejaculation	N/A	N/A	N/A	N/A	41	1.7	0.0	0–12.6
#5 Relationships	48	6.6	0.0	0–72.8	37	2.5	0.0	0–25.2
#6 My body	43	5.2	0.0	0–42.8	24	1.3	0.0	0–16.3

### 3.3 Effects of the intervention

#### 3.3.1 Primary outcome

No significant differences between the two groups were found for the domain “Satisfaction with sex life” at T1, or at T2 (see Table 4).

**Table 4:** Primary outcome, ‘Satisfaction with sex life’ at baseline (T0), post-intervention (T1), and at 3-month follow-up (T2).

Group	T0		T1			T2		
	Mean	*P* value	Mean (95 % CI)	P value	Cohen's d	Mean (95 % CI)	P value	Cohen's d
IG	44.15 *n* = 138	0.990	45.15 (43.56–46.74) *n* = 100	0.635	−0.065	45.50 (43.78–47.22) *n* = 86	0.264	−0.165
CG	44.16 *n* = 130		44.65 (43.24–46.05) *n* = 111			44.17 (42.55–45.78) *n* = 99		

IG: intervention group.

CG: control group.

In subgroup analyses, participants in both groups who reported greater dissatisfaction with their sex life (≥1 SD from the mean) at baseline (IG, *n* = 57; CG, *n* = 58) improved over time and were more satisfied at T1 and T2 (*p* ≤0.001), see Supplementary Fig. A. However, no significant effects were found based on participants' activity level.

#### 3.3.2 Secondary outcomes

The IG reported significantly more vaginal lubrication compared to the CG at T1 (*p* = 0.048, d= − 0.349), and these improvements were maintained at T2 (*p* = 0.023, d= − 0.446) (Table 5). Furthermore, the IG rated less emotional distress at T1 (*p* = 0.047, d=0.265). Sensitivity analyses revealed that these improvements were due to baseline levels, rather than group allocation. No significant effects were found in any of the remaining secondary outcomes.

**Table 5:** Differences in mean scores of secondary outcomes between the intervention group (IG) and the control group (CG).

Outcomes	T0 (baseline)			T1 (post-intervention)				T2 (3-months follow-up)			
	IG Mean (SD)	CG Mean (SD)	P value	IG Mean (SD)	CG Mean (SD)	P value	Cohen's d	IG Mean (SD)	CG Mean (SD)	P value	Cohen's d
Interest in sexual activity^a^, ↑	45.76 (10.92)	46.76 (10.26)	0.336	45.01 (11.50)	45.22 (10.63)	0.882	0.020	44.90 (11.20)	45.24 (10.85)	0.826	0.031
	n = 142	*n* = 135		n = 111	*n* = 118			*n* = 101	*n* = 104		
Orgasm – ability^a^, ↑	48.00 (12.52)	48.70 (9.97)	0.618	48.23 (12.27)	47.55 (10.04)	0.662	−0.061	50.76 (10.04)	48.61 (9.92)	0.156	−0.216
	*n* = 132	*n* = 127		*n* = 98	*n* = 110			*n* = 80	*n* = 96		
Orgasm – pleasure^a^, ↑	46.82 (8.58)	46.13 (9.02)	0.544	45.99 (8.03)	46.70 (8.40)	0.547	0.087	46.19 (7.88)	46.17 (8.41)	0.989	−0.002
	*n* = 120	*n* = 125		*n* = 90	n = 104			n = 80	*n* = 92		
Erectile function^a^, ↑	46.01 (8.32)	44.76 (8.67)	0.440	46.85 (8.32)	49.90 (8.01)	0.101	−0.371	47.57 (7.76)	47.08 (8.08)	0.797	−0.061
	n = 59	*n* = 53		*n* = 38	*n* = 42			*n* = 34	*n* = 37		
Vaginal lubrication^a^, ↑	47.25 (8.30)	44.94 (9.13)	0.103	47.65 (8.55)	44.53 (9.22)	**0.048**	−0.349	49.09 (9.17)	44.75 (10.22)	**0.023**	−0.446
	*n* = 78	*n* = 75		*n* = 62	*n* = 69			*n* = 50	n = 58		
Vaginal discomfort^a^, ↓	51.64 (8.70)	55.12 (9.54)	**0.021**	52.38 (8.70)	53.81 (10.16)	0.393	0.151	50.19 (8.28)	52.82 (9.05)	0.124	0.302
	*n* = 77	n = 73		n = 62	*n* = 68			n = 50	*n* = 56		
Vulvar discomfort – labial^a^, ↓	55.43 (9.25)	54.56 (8.54)	0.549	53.44 (8.71)	53.45 (8.60)	0.999	0.000	51.76 (7.10)	53.11 (8.28)	0.362	0.174
	n = 77	n = 75		*n* = 61	n = 69			n = 50	n = 59		
Vulvar discomfort – clitoral^a^, ↓	53.95 (8.77)	56.28 (9.43)	0.117	53.64 (8.73)	54.72 (9.60)	0.506	0.117	53.12 (8.04)	55.35 (10.26)	0.206	0.238
	n = 77	n = 75		n = 61	n = 69			n = 50	*n* = 60		
Body Image Scale↑	9.73 (6.95)	9.88 (6.98)	0.864	9.60 (6.88)	10.37 (7.56)	0.424	0.105	8.88 (6.48)	9.60 (7.06)	0.449	0.105
	n = 142	n = 135		*n* = 113	n = 118			n = 101	*n* = 106		
Hospital Anxiety Depression Scale↓	13.61 (7.91)	14.85 (8.39)	0.208	12.70 (7.29)	14.73 (8.03)	**0.047**	0.265	12.81 (7.94)	14.21 (7.73)	0.100	0.179
	*n* = 142	n = 136		n = 111	*n* = 116			n = 101	n = 104		
EORTC QLQ-C30^b^, ↑	74.51 (16.80)	74.83 (17.85)	0.881	76.77 (14.32)	75.22 (16.96)	0.465	−0.099	78.06 (14.51)	75.79 (16.93)	0.312	−0.144
	n = 138	n = 132		n = 104	n = 113			n = 96	*n* = 103		
Self-efficacy sex↑	3.19 (0.71)	3.23 (0.63)	0.681	3.31 (0.62)	3.19 (0.67)	0.084	−0.187	3.32 (0.61)	3.24 (0.62)	0.176	−0.133
	n = 135	*n* = 123		n = 106	n = 113			n = 96	n = 101		

**Note**. The number of observations differ between domains due to some domains being body-part specific.

a PROMIS SexFS domains.

b EORTC Quality of Life Questionnaire.

↑ Higher mean values indicate better sexual function, body image, health-related quality of life and self-efficacy sex.

↓ Lower mean values indicate better sexual function, less anxiety and depression.

In subgroup analyses, participants with greater sexual problems at baseline, in both the IG and the CG, improved over time in six of the nine selected SexFS domains (*p* ≤ 0.001–0.005), see Supplementary Fig. B-I. With regard to erectile function, an interaction effect was only found in the CG at T1 and T2 (*p* = 0.001). The IG also improved in erectile function, but not significantly (T1: *p* = 0.273, T2: *p* = 0.077). No significant differences in any of the SexFS domains were found at T1 or T2 based on participants' activity level in the intervention.

### 3.4 Self-perceived change in sexual problems after the intervention

Most of the participants in the IG (95/112, 85 %) completed a single item on self-perceived changes of sexual problems following the intervention (T1). The majority (77 %) of these reported that their problems had not changed, and one fifth (21 %) stated that their problems had been alleviated. Of those reporting a positive change, all but one had a high activity level in the intervention. Two participants (2 %) experienced a worsening of their problems, but gave no details.

In total, 29 participants provided a comment about their sexuality and sexual problems in the open-ended question at the end of the intervention. Responses included not perceiving oneself as having sexual problems before participation in the intervention (*n* = 10), while others had experienced increased understanding and acceptance of their sexual problems (*n* = 9). Furthermore, some participants stated that it takes more time to alleviate one's sexual problems, or that they thought they had used the intervention too little to achieve changes (*n* = 7).

## 4 Discussion

This RCT tested the efficacy of the psychoeducational web-based intervention, Fex-Can Sex, to reduce sexual problems in Swedish young adult survivors of childhood cancer. Overall adherence to the intervention was low and the intervention did not show any short-term effect on the primary outcome (Satisfaction with sex life). Our secondary outcomes showed that the IG, compared to the CG, reported more lubrication and less emotional distress following participation in the intervention. However, these results were not significant when controlling for baseline levels. Furthermore, participants with high levels of sexual dysfunction at baseline improved over time in several SexFS domains, with similar patterns in both groups (IG and CG). Increased understanding and acceptance of their sexual problems were also reported by some IG participants.

Despite that no significant effects of the intervention were found based on participants' activity level, we believe that the limited effects detected between the IG and the CG could be related to the overall low activity in the intervention. Adherence is a common challenge in web-based interventions (Sieverink et al., 2017), particularly in self-guided interventions (Karyotaki et al., 2015). Previous research suggests that low adherence may have a negative impact on the effectiveness of an intervention (Donkin et al., 2011; Eysenbach, 2005). In our study, most members of the IG spent <20 min on the entire 12-week intervention and opened half or fewer of the modules included in the programme. This low adherence is assumed to be related to how participants were recruited. Study participants were identified in a large population-based sample of childhood cancer survivors and all individuals who rated sexual dysfunction according to a pre-defined threshold were approached (Hovén et al., 2021; Ljungman et al., 2020). Such a recruitment strategy, i.e. not self-referral, combined with the low threshold for sexual dysfunction (0.5 SD from the mean), risks including people who are less troubled by sexual problems. This was confirmed by some participants, who spontaneously commented that they had consented to participate in the study because they wanted to help others. Additionally, our definition of high activity (spending ≥20 min over the 12-week period, opening at least half of the modules, and using one of the interactive components) may have been set too low to achieve improvements, as we do not see any effects in the study outcomes.

Furthermore, we did not investigate the duration of participants' problems related to sexuality and intimacy. This should be taken into consideration because sexual problems are complex, involve both physical and psychosocial factors, and often take longer than 12 weeks to resolve, especially if the problems have been present for several years. The limited effects of the intervention should also be considered in the context of Swedish childhood cancer survivors, both women and men, being partnered to a lesser extent than the general population (Hovén et al., 2021), which was also seen in this RCT. Previous web-based interventions that have reported positive effects on sexual problems have been based on samples who were partnered to a greater extent (Hummel et al., 2017; Schover et al., 2012; Schover et al., 2020; Wootten et al., 2017) than our sample.

Somewhat unexpectedly, no differences in any outcomes were found by level of activity. One would expect that those who spent more time on the intervention would show greater improvements. However, it is questionable whether the definition of high activity (≥20 min in the programme, ≥3 modules opened, ≥1 interaction criteria) corresponds to a level of activity that can produce an independent effect, given the overall low activity in the intervention.

Our findings of low adherence are in line with those of some previous studies (Micaux et al., 2022; Schover et al., 2012). However, Hummel et al. (2017) tailored the content of their intervention to meet each participant's needs, which were pre-assessed before start, and found that more than half of their participants completed the intervention (62 %). Furthermore, their results showed positive effects on several aspects of sexual dysfunction (Hummel et al., 2017). Addressing participants' individual needs may increase their motivation to engage with and adhere to the intervention, thereby leading to greater mitigating of sexual problems.

In line with a previous study (Schover et al., 2012), we found improvements in sexual function for those who had greater sexual problems at baseline. However, we cannot fully explain these results because the same pattern is seen for both groups (IG and CG). For those who have more sexual problems there is greater room for improvement, which results in an increased likelihood of detecting effects, compared to those who do not perceive themselves as having any sexual problems. We don't know if participants in the CG sought information or support in other ways to deal with their sexual problems, and therefore improved.

Although few statistically significant differences were found, an increased understanding and acceptance of their sexual problems were reported by some members of the IG. This finding is in line with an interview study addressing young adults (*n* = 28, aged 19–40) with cancer following participation in a similar web-based psychoeducational intervention targeting both sexual dysfunction and fertility-related distress (Micaux Obol et al., 2020). The researchers concluded that intervention participants improved their understanding of cancer-related facts and their own reactions, and that they felt more supported and less lonely. Some participants expressed that they did not associate their sexual problems with their former cancer diagnosis. Sexuality and possible negative impact on sexual function is recommended to be addressed during follow-up care.

Overall, our intervention showed few differences between the IG and CG on sexual problems. As the inclusion criteria for sexual dysfunction were set at a rather low cut-off, we believe that a substantial proportion of the participants did not experience their sex life as especially problematic, which was also reflected in the open-ended comments. This can probably explain the low adherence as well as the small effects. Therefore, we intend to further develop the Fex-Can programme to increase uptake and adherence. The inclusion criteria should ensure that the individuals included are troubled by sexual problems, a more tailored content based on the participants' specific problems and more interaction are believed to improve adherence and the effects of the programme.

### 4.1 Methodological considerations

This study has several strengths including the randomized controlled design, the large sample size and high response rates throughout the study, as well as the use of validated instruments. Furthermore, the co-creation with former cancer patients engaged in the development of the intervention. However, low adherence to the intervention makes it difficult to draw firm conclusions. Unfortunately, the reasons for low adherence were not investigated. Nor do we know whether participants sought information and/or support in other ways during the period of the intervention and follow-up which may have had an impact on the results.

### 4.2 Conclusion

Despite the low adherence to the Fex-Can Sex intervention and limited effects overall, there were notable improvements among participants with greater sexual dysfunction at baseline. We recommend the intervention and the study procedures to be further developed, including the use of stricter inclusion criteria, to include those who are troubled by sexual problems and willing to engage in an intervention, and more tailored content to improve adherence and effect.

The following are the supplementary data related to this article.

Supplementary Fig. A Interaction effect (level of sexual dysfunction at baseline ∗ time), linear mixed models with subject specific random intercept in the SexFS domain ‘Satisfaction with sex life’ (primary outcome) at post-intervention (T1) and 3-months follow-up (T2) compared to baseline (T0). Supplementary Fig. A

Supplementary Fig. B-I Interaction effect (level of sexual dysfunction at baseline ∗ time), linear mixed models with subject specific random intercept in the selected SexFS domains (secondary outcomes) at post-intervention (T1) and 3-months follow-up (T2) compared to baseline (T0). Supplementary Fig. B-I

## Funding

This study was funded by the 10.13039/501100002794Swedish Cancer Society (CAN 2013/886, CAN 2016/615, 190196Pj); the 10.13039/501100006313Swedish Childhood Cancer Foundation (TJ2014-0050, TJ2019-0045, PR2014-0177, PR2016-0075, and PR2017-0037, KP 2020-0012); the 10.13039/501100006636Swedish Research Council for Health, Working Life and Welfare (2014-4689, 2019-00839); the 10.13039/501100004359Swedish Research Council (2017-01530), the Karolinska Institutet Faculty Funds (2-5586/2017); the Birgitta and Carl-Axel Rydbeck's Research Grant for Paediatric Research (2021-00079); and the Centre for Clinical Research Sörmland (10.13039/501100007051Uppsala University). The funding sources were not involved in the design or conduction of the study.

## CRediT authorship contribution statement

**Conceptualisation**: Lampic, Wettergren, Fagerkvist; **Data curation**: Fagerkvist, Jahnukainen, Lampic, Wettergren; **Formal analysis**: Fagerkvist; **Funding acquisition**: Lampic, Wettergren, Jahnukainen, Fagerkvist; **Investigation**: Fagerkvist, Lampic, Wettergren; **Methodology**: Ljungman, Lampic, Wettergren, Fagerkvist; **Project administration**: Lampic, Wettergren, Fagerkvist; **Resource**: Lampic, Wettergren; **Supervision**: Lampic, Wettergren; **Visualisation**: Fagerkvist, Jahnukainen, Lampic, Ljungman, Wettergren; **Writing** – **original Draft**: Fagerkvist; **Writing** – **Review and editing**: Fagerkvist, Jahnukainen, Lampic, Ljungman, Wettergren.

## Declaration of competing interest

The authors declare that they have no known competing financial interests or personal relationships that could have appeared to influence the work reported in this paper.
