# Supplementary material for: Efficacy of a web-based psychoeducational intervention, Fex-can sex, for young adult childhood cancer survivors with sexual dysfunction: A randomized controlled trial
Source: Internet Interv. 2024 Apr 7;36:100739. doi: 10.1016/j.invent.2024.100739 (PMC11016752; doi:10.1016/j.invent.2024.100739)
Supplement: Supplementary Fig. A — Interaction effect (level of sexual dysfunction at baseline ∗ time), linear mixed models with subject specific random intercept in the SexFS domain ‘Satisfaction with sex life’ (primary outcome) at post-intervention (T1) and 3-months follow-up (T2) compared to baseline (T0). [file mmc1.docx]

**Supplementary Figure A.** Interaction effect (level of sexual dysfunction at baseline*time), linear mixed models with subject specific random intercept in the SexFS domain ‘Satisfaction with sex life’ (primary outcome) at post-intervention (T1) and 3-months follow-up (T2) compared to baseline (T0).


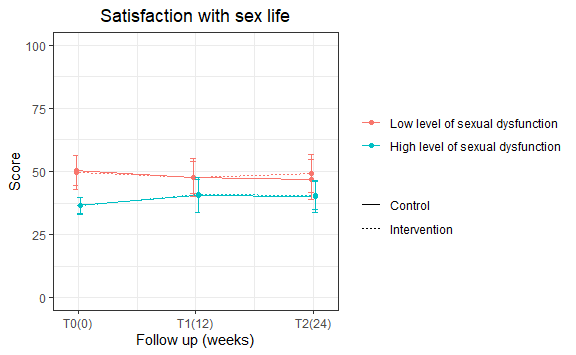


**Figure A**. Satisfaction with sex life, high level of sexual dysfunction (≥1 SD below the mean) at and compared to T0. **IG**: T1 (n=35) + T2 (n=32), p=<0.001. **CG**: T1 (n=34) + T2 (n=29) p=<0.001
