# Supplementary material for: Efficacy of a web-based psychoeducational intervention, Fex-can sex, for young adult childhood cancer survivors with sexual dysfunction: A randomized controlled trial
Source: Internet Interv. 2024 Apr 7;36:100739. doi: 10.1016/j.invent.2024.100739 (PMC11016752; doi:10.1016/j.invent.2024.100739)
Supplement: Supplementary Fig. B-I — Interaction effect (level of sexual dysfunction at baseline ∗ time), linear mixed models with subject specific random intercept in the selected SexFS domains (secondary outcomes) at post-intervention (T1) and 3-months follow-up (T2) compared to baseline (T0). [file mmc2.docx]

**Supplementary Figures B-I.** Interaction effect (level of sexual dysfunction at baseline*time), linear mixed models with subject specific random intercept in the selected SexFS domains (secondary outcomes) at post-intervention (T1) and 3-months follow-up (T2) compared to baseline (T0).


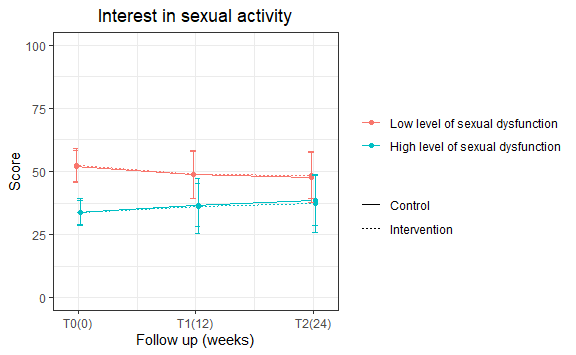


**Figure B.** Interest in sexual activity, high level of sexual dysfunction (≥1 SD below the mean) at and compared to T0. **IG**: T1 (n=37) + T2 (n=34) p=<0.001. **CG**: T1 (n=43) p=0.002, T2 (n=42) p=<0.001.


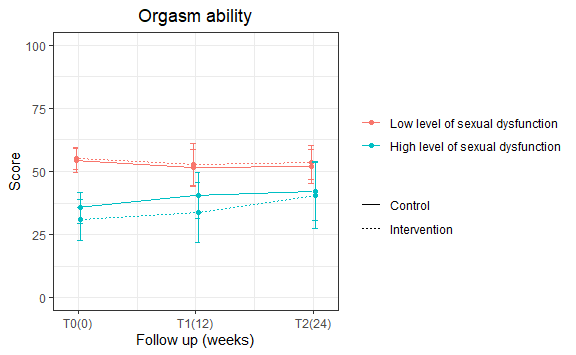


**Figure C.** Orgasm ability, high level of sexual dysfunction (≥1 SD below the mean) at and compared to T0. **IG**: T1 (n=23) p=0.004, T2 (n=17) p=<0.001. **CG**: T1 (n=34) + T2 (n=30) p=<0.001.


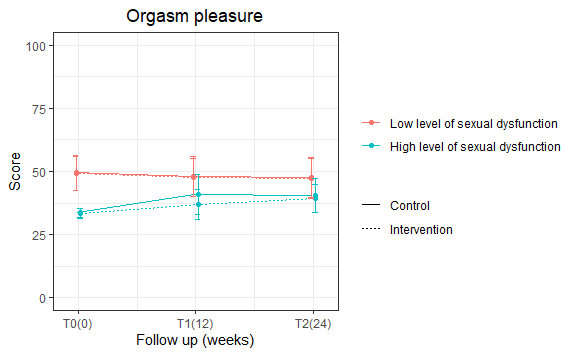


**Figure D.** Orgasm pleasure, high level of sexual dysfunction (≥1 SD below the mean) at and compared to T0. **IG:** T1 (n=9) p=0.005, T2 (n=12) p=<0.001. **CG:** T1 (n=21) +T2 (n=19) p=<0.001.


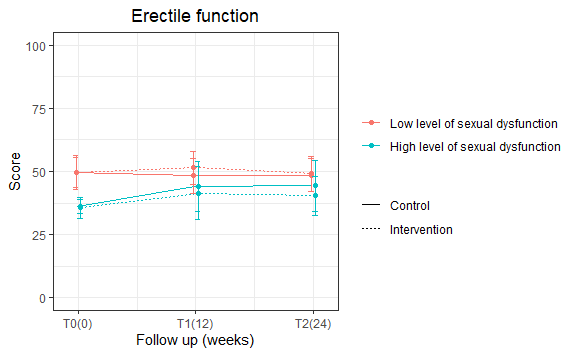


**Figure E.** Erectile function, high level of sexual dysfunction (≥1 SD below the mean) at and compared to T0. **IG:** T1 (n=6) p=0.273, T2 (n=6) p=0.077. **CG:** T1 (n=16) + T2 (n=13) p=0.001.


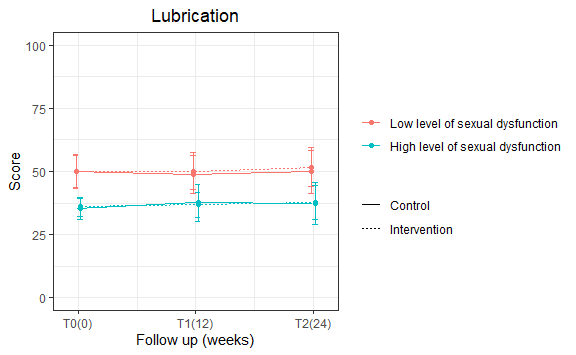


**Figure F.** Lubrication, high level of sexual dysfunction (≥1 SD below the mean) at and compared to T0. **IG:** T1 (n=11) p=0.947, T2 (n=9) p=0.949. **CG:** T1 (n=23) p=0.018, T2 (n=23) p=0.122.


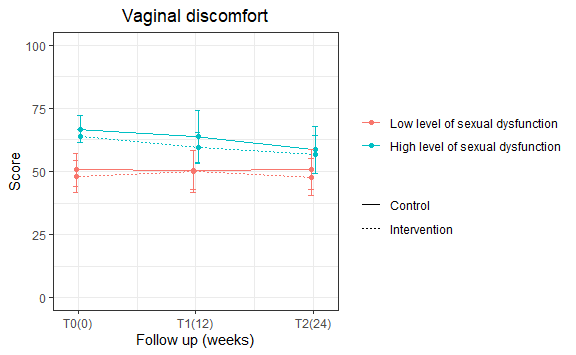


**Figure G.** Vaginal discomfort, high level of sexual dysfunction (≥1 SD above the mean) at and compared to T0. **IG:** T1 (n=15) p=0.011, T2 (n=13) p=0.007. **CG:** T1 (n=17) p=0.250, T2 (n=16) p=0.002.


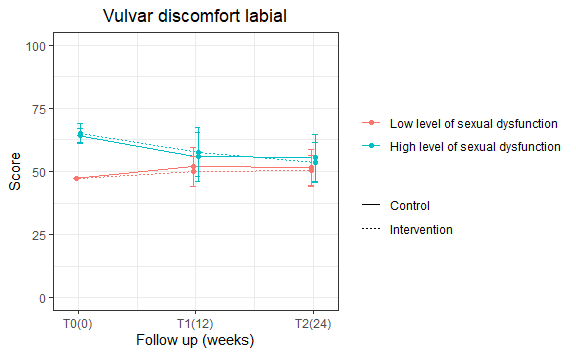


**Figure H.** Vulvar discomfort labial, high level of sexual dysfunction (≥1 SD above the mean) at and compared to T0. **IG:** T1 (n=28) + T2 (n=23) p=<0.001. **CG:** T1 (n=28) + T2 (n=26) p=<0.001.


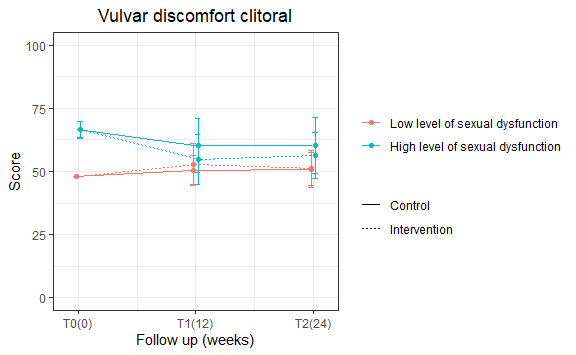


**Figure I.** Vulvar discomfort clitoral, high level of sexual dysfunction (≥1 SD above the mean) at and compared to T0. **IG:** T1 (n=18) + T2 (n=15) p=<0.001. **CG:** T1 (n=28) + T2 (n=29) p=<0.001.
